# A 58‐year‐old man with B‐cell chronic lymphocytic leukemia and multiple strokes

**DOI:** 10.1111/bpa.13004

**Published:** 2021-07-30

**Authors:** Francesca Magrinelli, Sara Mariotto, Gianpaolo Nadali, Giuseppe Todeschini, Massimiliano Lanzafame, Tiziana Cavallaro, Salvatore Monaco, Sergio Ferrari

**Affiliations:** ^1^ Department of Neurosciences, Biomedicine and Movement Sciences University of Verona Verona Italy; ^2^ Department of Medicine University of Verona Verona Italy; ^3^ Department of Diagnostic and Public Health University of Verona Verona Italy

**Keywords:** Aspergillosis, brain, immunodeficiency, mycosis, neuropathology

## Abstract

A 58‐year‐old male with B‐cell chronic lymphocytic leukemia presented with fever, chest pain, and acute‐onset neurological deficits suggestive of multiple strokes (A). Brain autopsy revealed softening areas in the brain parenchyma (B, C) corresponding to extensive necrosis (D) caused by neuroinvasion by *Aspergillus* hyphae (E, F) necrosis (D) caused by neuroinvasion by *Aspergillus* hyphae (E, F).

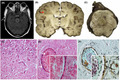

BOX 1 **Slide scan**

Access the whole slide scan at http://image.upmc.edu:8080/NeuroPathology/BPA/BPA‐21‐01‐009/view.apml

## 1 CASE PRESENTATION

**FIGURE 1:**
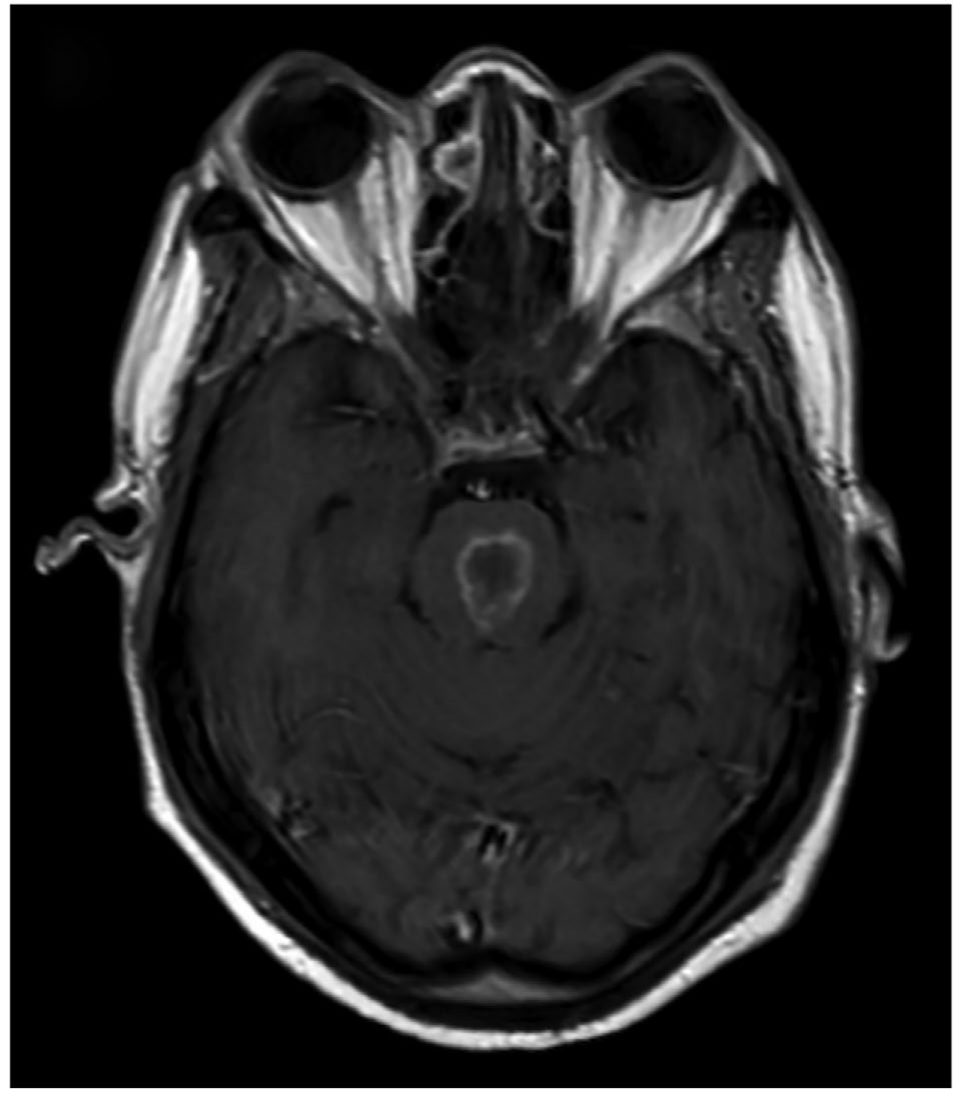
Axial, gadolinium‐enhanced, T1‐weighted MRI image showing abscessual evolution of a brain lesion in the pons 9 days after the onset of neurological symptoms

A 58‐year‐old African male presented with sudden‐onset slurred speech after few days of mild fever and diffuse chest pain. He had a 7‐year history of B‐cell chronic lymphocytic leukemia, which was treated with multiple lines of chemotherapy during relapses and was recently suspected of transformation into large B‐cell lymphoma. He was on low‐dose prednisone and ibrutinib. His body temperature was 37.2℃, and neurological examination revealed dysarthria and right‐sided central facial palsy. C‐reactive protein was 204 mg/L (reference range, <5), white blood cell count 15,390/mm^3^ (reference range, 4300–10,000) with lymphocytosis and neutropenia (610 neutrophils/mm^3^; reference range, 2000–7000), and platelet count 41,000/mm^3^ (reference range, 150,000–400,000). Brain computed tomography (CT) showed a subtle focus of hypodensity involving the left frontal lobe. Chest radiography detected bilateral lung nodules. His neurological picture rapidly worsened with right‐sided weakness and numbness, motor aphasia, internuclear ophthalmoplegia, and dysphagia. Brain magnetic resonance imaging (MRI) revealed DWI‐positive lesions (with low ADC values) in the frontal lobes and pons, one of which showed minimal peripheral contrast enhancement. QuantiFERON‐TB Gold, and Toxoplasma, and human immunodeficiency virus (HIV) serology were negative. Cerebrospinal fluid (CSF) analysis, including bacterial and fungal cultures and PCR for neurotropic viruses, was unremarkable. CSF flow cytometry revealed T and rare B19+ lymphocytes. The patient was started on levofloxacin, piperacillin‐tazobactam, and voriconazole, whereas ibrutinib and prednisone were discontinued. Chest CT revealed bilateral pulmonary nodular infiltrates surrounded by foci of ground‐glass opacities. Serum and CSF (1,3)‐β‐D‐glucan and galactomannan tests were positive, while CSF and serum cryptococcal antigen and CSF VDRL were negative. The patient's level of consciousness slowly deteriorated during the following weeks. On follow‐up MRI nine days after neurological symptom onset, brain lesions previously detected showed ring enhancement in post‐gadolinium T1‐weighted sequences (Figure 1). The patient died three weeks after admission.

## 2 FINDINGS

**FIGURE 2:**
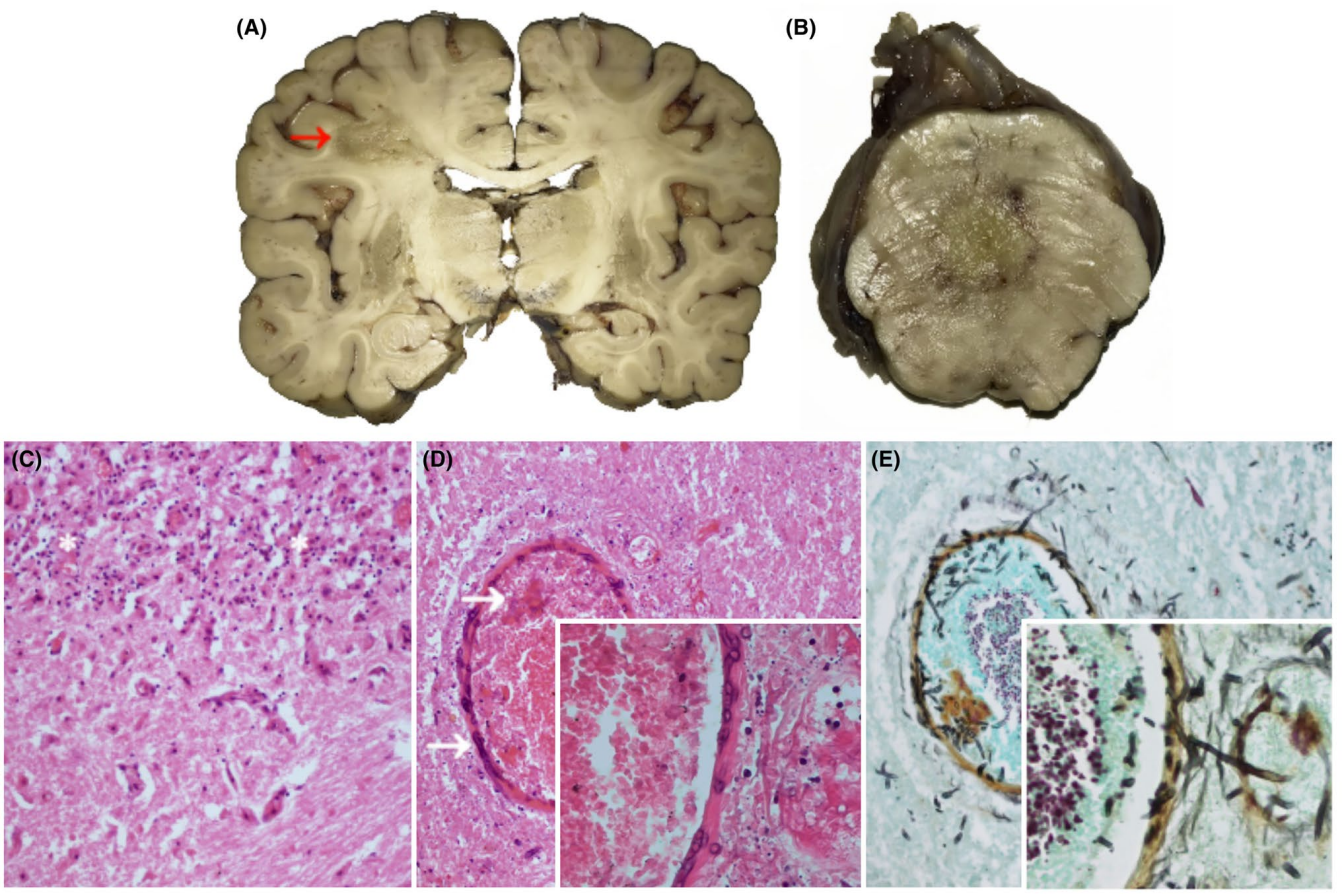
(A) Coronal slice of cerebral hemispheres revealing a yellowish softening subcortical area in the frontal lobe (red arrow). (B) Round softening area with peripheral haemorrhagic areolae in a transverse section of the central pons. (C) Hematoxylin‐eosin staining of the central pons showing extensive necrosis with infiltration by polymorphonuclear leukocytes and macrophages (asterisks) in the brain parenchyma surrounding a blood vessel (original magnification ×10). (D) Numerous tubule‐like structures, consistent with *Aspergillus* hyphae, are scattered in the wall of a cerebral blood vessel (arrow) admixed with thrombus in the lumen (arrow) (hematoxylin‐eosin staining; original magnification ×20); inset: demonstration of fungal hyphae (original magnification ×40). (E) On methenamine silver stain, hyphae appeared as narrow, septate filaments with acute‐angle branching laying in the arteriolar wall and lumen and extending into adjacent necrotic tissue (original magnification ×20); inset demonstration of fungal hyphae (original magnification ×40)

On gross examination of the formalin‐fixed brain, meninges showed milky opacity with thickening mainly detectable at the base. There were no signs of intracranial hypertension. A small calcified plaque was observed at the basilar artery. Coronal slices of cerebral hemispheres revealed yellowish softening subcortical areas in the frontal lobes (Figure 2A, arrow). Another round softening area with peripheral hemorrhagic areolae was detected in transverse sections of the central pons (Figure 2B). There were no lesions in the cerebellum. After hematoxylin‐eosin staining of paraffin‐embedded brain sections, the softened lesions showed an extensive necrotic core surrounded by the proliferation of polymorphonuclear leukocytes, multinucleated cells, and macrophages (Figure 2C, asterisks) positive for CD68. Faintly visible tubule‐like structures were scattered in the wall of cerebral blood vessels and admixed with thrombotic material into their lumen (Figure 2D, arrows). On methenamine silver stain, they appeared as narrow, septate filaments with acute‐angle (~45°) branching laying in the arteriolar wall and lumen and extending into adjacent necrotic tissue (Figure 2E). Meninges were modestly infiltrated by lymphocytes and macrophages.

## 3 DIAGNOSIS

Invasive aspergillosis with central nervous system (CNS) localizations.

## 4 DISCUSSION

*Aspergillus* spp. are ubiquitous airborne molds in indoor and outdoor environments. Few species, including *Aspergillus fumigatus*, *Aspergillus flavus*, *Aspergillus niger*, and *Aspergillus terreus*, cause a spectrum of clinical syndromes in humans. Invasive aspergillosis (IA) is the most severe and life‐threatening presentation of aspergillosis in hematological and solid organ transplant patients, whereas it is relatively uncommon in HIV‐positive patients (1). In at‐risk populations, predisposing factors include prolonged and severe neutropenia, prolonged therapy with a high‐dose corticosteroid, and chemotherapy‐induced T‐cell depletion. Lung is usually the site of primary infection, from which the pathogen may spread to nearly any organ via the hematogenous route. Less commonly, IA starts in other sites, such as sinuses, gastrointestinal tract, or skin. As a result of their marked vascular tropism, *Aspergillus* spp. hyphae can penetrate vessel walls and produce necrotizing vasculitis. CNS involvement by *Aspergillus* spp. (mainly *A*. *fumigatus* and *A*. *flavus*) typically results in cerebral infarction with or without hemorrhage and brain abscess formation, whereas meningitis occurs more rarely (1). Manifestations of cerebral aspergillosis are often non‐specific, including fever, seizures, alteration of mental status, and stroke‐like events. The clinical picture mainly depends on the location, extent, and character of the lesions. Early diagnosis of IA is challenging and should be based on the integration of clinico‐radiological and microbiological data (1). Typical findings on chest CT include multiple nodules and the halo sign, a zone of low attenuation because of hemorrhage surrounding pulmonary nodules. If the CNS is involved, brain MRI typically shows ring‐enhanced lesions, infarction, and vascular infiltration from adjacent lesions (2). Serum and CSF biomarkers, such as galactomannan antigen and (1,3)‐β‐D‐glucan, are useful for early diagnosis as fungal cultures are often unremarkable in IA (1). Histopathological examination of lung or other tissues revealing septate hyphae along with a culture positive for *Aspergillus* spp. from the same site is the diagnostic gold standard. In our case, post‐mortem neuropathology revealed non‐pigmented septate hyphae with Y‐shaped branching, which were disseminated in the wall and lumen of blood vessels, and at the periphery of necrotic brain parenchyma. As *Aspergillus* spp. hyphae share these morphological features with other hyaline septate molds having different antifungal susceptibility (e.g., *Fusarium* spp., *Scedosporium* spp.), advanced techniques (e.g., immunohistochemistry, *in situ* hybridization, PCR) should be applied to identify the specific organism (3). Interestingly, cultures for *Fusarium* spp. have a high yield compared to those for *Aspergillus* spp. because of their adventitious sporulation in tissues (3). Hyphal elements with necrosis, hemorrhage, and blood vessel thrombosis may also be detected in CNS mucormycosis. However, *Mucorales* genera produce non‐pigmented, ribbon‐like, non‐septate, or pauci‐septate hyphae that exhibit right‐angle branching, and the application of previously cited techniques to differentiate these species from *Aspergillus* spp. and other septate molds may be necessary in challenging cases (3). As (1,3)‐β‐d‐glucan and galactomannan tests do not detect antigen components of the *Mucorales*’ cell wall, the positivity of these tests provides strong evidence for excluding *Mucormycetes* as causative agents of infection. Prompt antifungal therapy initiation is critical for the outcome. Voriconazole and isavuconazole are drugs of choice in IA with CNS involvement. Alternatives are high doses of liposomal amphotericin B, while data on the efficacy of echinocandins are limited (1). Despite advances in early diagnosis and treatment of disseminated fungal infections, the mortality rate of IA with CNS involvement in immunocompromised hosts still exceeds 90% (1).

## CONFLICT OF INTEREST AND DISCLOSURES

The authors have no conflict of interest and disclosures to declare.

## AUTHOR CONTRIBUTIONS

Francesca Magrinelli: study design, acquisition of data, drafting the manuscript, acquisition of data. Sara Mariotto, Gianpaolo Nadali, Giuseppe Todeschini, Massimiliano Lanzafame, Tiziana Cavallaro, Salvatore Monaco: acquisition of data, revising the manuscript. Sergio Ferrari: study design, acquisition of data, interpretation of pathological results, revising the manuscript.

## Data Availability

Data sharing not applicable to this article as no datasets were generated or analyzed during the current study.
